# No Evidence that MDMA-Induced Enhancement of Emotional Empathy Is Related to Peripheral Oxytocin Levels or 5-HT_1a_ Receptor Activation

**DOI:** 10.1371/journal.pone.0100719

**Published:** 2014-06-27

**Authors:** Kim P. C. Kuypers, Rafael de la Torre, Magi Farre, Samanta Yubero-Lahoz, Isabel Dziobek, Wouter Van den Bos, Johannes G. Ramaekers

**Affiliations:** 1 Dept. of Neuropsychology & Psychopharmacology, Fac. of Psychology & Neuroscience, Maastricht University, Maastricht, the Netherlands; 2 IMIM- Hospital del Mar- Research institute, Human Pharmacology & Clinical Neurosciences Research Group, Barcelona, Spain; 3 Cluster of Excellence Languages of Emotion, Freie Universität, Berlin, Germany; 4 Center for Adaptive Rationality (ARC), Max-Planck-Institute for Human Development, Berlin, Germany; University of Chicago, United States of America

## Abstract

The present study aimed at investigating the effect of MDMA on measures of empathy and social interaction, and the roles of oxytocin and the 5-HT_1A_ receptor in these effects. The design was placebo-controlled within-subject with 4 treatment conditions: MDMA (75 mg), with or without pindolol (20 mg), oxytocin nasal spray (40 IU+16 IU) or placebo. Participants were 20 healthy poly-drug MDMA users, aged between 18–26 years. Cognitive and emotional empathy were assessed by means of the Reading the Mind in the Eyes Test and the Multifaceted Empathy Test. Social interaction, defined as trust and reciprocity, was assessed by means of a Trust Game and a Social Ball Tossing Game. Results showed that MDMA selectively affected emotional empathy and left cognitive empathy, trust and reciprocity unaffected. When combined with pindolol, these effects remained unchanged. Oxytocin did not affect measures of empathy and social interaction. Changes in emotional empathy were not related to oxytocin plasma levels. It was concluded that MDMA (75 mg) selectively enhances emotional empathy in humans. While the underlying neurobiological mechanism is still unknown, it is suggested that peripheral oxytocin does not seem to be the main actor in this; potential candidates are the serotonin 2A and the vasopressin 1A receptors.

**Trial Registration:**

MDMA & PSB NTR 2636

## Introduction

Almost 30 years ago, MDMA's prosocial effects (e.g. heightened closeness) were documented for the first time in recreational users [1]. Since then, more studies have been conducted, mostly by means of self-reports or questionnaires in ecstasy users, either under influence or in a sober or abstinent state (e.g. [2], [3]). Findings showed that MDMA induced subjective feelings of closeness to, and openness towards others, emotional warmth, enhanced well-being, contentment, empathy and euphoria (e.g. [4], [5]). Dumont and colleagues (2009) for example used two items from the Bond and Lader Mood Rating Questionnaire to explore ‘prosocial states’ and showed an MDMA-induced increase in self-rated amicability and gregariousness compared with placebo. Kirkpatrick and colleagues (2014) showed that participants under influence of MDMA (1.5 mg/kg) were more likely to rate socializing with other as more desirable compared to placebo [6].

Only relatively recent, studies are being published using objective measures of social behavior and cognition in addition to subjective measures, to quantify the prosocial effects of MDMA. The first to cover this were Bedi and colleagues (2009) who conducted a pharmaco-imaging study into the effects of MDMA on emotion recognition (defined here as ‘cognitive empathy’; to differentiate ‘knowing’ from ‘feeling’). MDMA (0.75 & 1.5 mg/kg) increased activation in the ventral striatum in response to happy faces and decreased amygdala activation in response to angry faces (only at 1.5 mg/kg) [4]. In another study, Bedi and colleagues (2010) showed a selective reduction in recognition of fearful faces after MDMA (1.5 mg/kg) administration in the ‘Face Emotion Recognition Task’ (FERT), while recognition of other emotions (angry, happy, neutral, sad) was left unaffected [7]. A few years later, the same group showed that MDMA (1.5 mg/kg) impaired recognition of negative facial expressions (anger and fear) in the ‘Morphed Facial Expression Task’, a task comparable to the FERT [6]. In line with Bedi et al. (2010) [7], Hysek and colleagues (2012) also showed an absence of MDMA (125 mg) effects on emotion recognition as measured by another task, i.e. the ‘Reading the Mind in the Eyes Test’ (RMET) [8]. However, when taking valence of the items into account, Hysek et al. (2012) found a dissociation in effects; i.e., MDMA enhanced recognition of positive emotions whereas recognition of negative emotions was impaired [8]. In another study, Hysek and colleagues (2013) showed absence of MDMA effects on cognitive empathy in one task (Multifaceted Empathy Task, ‘MET’) while revealing impairment of recognition of negative emotions in the FERT. This was contrasted with the MDMA-induced increase in emotional empathy (‘arousal’, ‘concern’) in the MET [9].

Taken together, evidence shows that MDMA enhances emotional components of empathy, as assessed by subjective and objective measures, and impairs cognitive empathy (emotion recognition) for negative emotions [4], [6]–[10]. The effect of MDMA on cognitive empathy for positive emotions however was less consistent and only shown in one study on one behavioral parameter [8] and in another study on a neuronal level [4].

Besides the relative dearth of research into the effect of MDMA on empathy, the use of objective measures of social interactions is even scarcer. Knowledge about the effects of MDMA on, for example, levels of trust giving and cooperativeness is important as this might underlie the hypothesized strengthening of the therapeutic alliance in MDMA-assisted psychotherapy [11], thereby facilitating the therapeutic process. To date, only two studies reported about this, i.e. Frye and colleagues (2014) who used a virtual ball tossing game and Hysek and colleagues (2013) who used a social value orientation task (SVO) [9], [12]. It was shown that MDMA (1.5 mg/kg) decreased the perceived objective level of rejection as experimentally created in the ball tossing game, and that MDMA (125 mg) increased prosocial behavior in the SVO task. Both studies indicate that MDMA enhances social interactions. The present study included a number of measures to assess the effects of MDMA on empathy (emotional and cognitive empathy) and social interaction (trust and reciprocity).

Another important issue to address is the neurochemical mechanism underlying the acute effects of MDMA on social behavior in humans. Recently, studies have focused on the neuropeptide oxytocin as a mediator in prosocial effects [3], [13]. Two studies that assessed oxytocin concentrations in blood after MDMA administration, both showed an MDMA-induced increase in those levels [3], [9]. Additionally, these concentrations were positively correlated with positive mood effects [3]. However, it was also shown that there was no correlation between plasma oxytocin concentrations and behavioral measures of empathy [9]. Kirkpatrick and colleagues (2014) compared the effects of intranasal oxytocin administration on mood and social cognition/behavior measures with MDMA effects. They showed that subjective responses after 40 IU oxytocin were not related to MDMA responses whereas those after 20 IU were [6]. Interestingly, oxytocin effects are partly mediated by the serotonin 5-HT_1A_ receptor [14]. The 5-HT_1A_ receptor is one of the main mediators of the action of serotonin, a neurotransmitter that acts as an intermediary for the main effects of MDMA [15]. Activation of this receptor has also been linked to an augmentation in sociability. Recent animal studies suggested that the 5-HT_1A_ receptor could play a key role in the prosocial effects of MDMA [16]. To date, no human research has been conducted into the effects of this receptor in MDMA-induced prosocial effects. However, Hasler and colleagues (2009) showed that pindolol, a beta blocker with 5-HT_1A_ receptor affinity has a minor modulating effect on subjective states, induced by MDMA [17]. Although the effects were small, pindolol seems to have the potential to mitigate MDMA-induced effects, and will therefore be used in the present study to study the role of the 5-HT_1A_ receptor in MDMA-induced effects.

The aim of the present study was to assess the effects of MDMA on different objective and subjective measures of empathy and social interaction, and to investigate the roles of oxytocin and the 5-HT_1A_ receptor in MDMA-induced prosocial effects. It was hypothesized that oxytocin and the 5-HT_1A_ receptor are key mediators of MDMA's prosocial effects and that 1) oxytocin would mimic MDMA-induced effects, and 2) blockade of the 5-HT_1A_ receptor would prevent occurrence of these effects when blocking was combined with MDMA intake.

## Materials and Methods

### Participants

Participants were 20 healthy poly-drug MDMA users, aged between 18–26 years, recruited through advertisements in university buildings and a website (digi-prik.nl), and by word of mouth. Demographic details are presented in Table 1.

**Table 1:** Demographic data of participants.

	Min	Max	Mean (SD)	N	
Age	18	26	21.60 (2.45)	20	
Verbal IQ	100	115	107.45 (4.41)	20	
	Males	Females	Total Mean (SD)	N	
Gender (N)	12	8		20	
Body Weight (Mean (SD))	78.00 (10.33)	58.62 (6.32)	70.25 (13.09)	20	
Drug use (Number of times used in lifetime)	Min	Max	Mean (SD)	N used	N never used
Ecstasy/MDMA	3	40	10.95 (8.97)	20	0
Amphetamine	1	25	12 (12.30)	4	16
Cannabis	1	240	67.18 (81.38)	16*	4
Cocaine	3	25	11 (9.76)	4	16
Mushrooms	1	4	2.17 (1.17)	6	14
LSD	0	0	0	0	20
Other: 2CB	1	1	-	1	19

*5 participants answered with: don't know (2X), since recently weekly, monthly, often.

### Design and Treatments

The study was conducted according to a 4-way placebo-controlled within-subject design. Three treatments i.e., pindolol capsules (20 mg) or placebo, MDMA capsules (75 mg) or placebo, and an oxytocin nasal spray (40 IU+2 booster doses of both 8 IU) or placebo spray, were combined to entail 4 Treatment Conditions. Every participant received 3 treatments (active or placebo) each test day in order to blind them for Treatment Condition (see Table 2): (1) pindolol capsule + MDMA capsule + placebo spray; (2) placebo capsule + MDMA capsule + placebo spray; (3) placebo capsule + placebo capsule + oxytocin spray; (4) placebo capsule + placebo capsule + placebo spray. Treatment Conditions were randomized.

**Table 2:** Schematic representation of a test day; BP =  Blood Pressure, HR =  Heart Rate; * T3 =  first dose of 40 IU.

Time	Time relative to T1	Time relative to T2	Time relative to T3*	Activity
9:00	−30′	−1 h30′	−2 h15′	GSS; POMS 1; BP/HR 1; Blood Samples 1
9:30	0	−1 h	−1 h45′	T1: Pindolol or Placebo
10:30	1 h	0	−45′	T2: MDMA or Placebo
11:15	1 h45′	45′	0	T3: Oxytocin or Placebo
11:55	2 h25′	1 h25′	40′	POMS 2; BP/HR 2; Blood Samples 2
12:00	2 h30′	1 h30′	45′	Test block 1: WLT-IR
12:10	2 h40′	1 h40′	55′	T3-booster 1 (8 IU)/placebo
12:15	2 h45′	1 h45′	1 h	Test block 2: Empathy (RMET, MET, IRI)
12:25	2 h55′	1 h55′	1 h10′	T3- booster 2 (8 IU)/placebo
12:30	3 h	2 h	1 h15′	Test block 3: Social interaction (Trust Game, SBTG& SBTG-questionnaire) and WLT-DR& Recognition
13:15	3 h45′	2 h45′	2 h	BP/HR 3 Blood Samples 3

Pindolol is a beta-blocker with affinity for the 5-HT_1A_ auto- and post-synaptic receptors [18]. Pindolol 20 mg has previously been shown to produce substantial 5-HT_1A_ occupancy (40%) at both the auto- and post-synaptic receptors, 2 hours after oral administration [18]. Ligands that selectively and fully block the 5-HT_1A_ receptor are not available. It was included in the present study to block the 5-HT_1A_ receptors.

MDMA (75 mg) has previously been shown to cause acute effects on mood and cognition (e.g. [19], [20]). Peak plasma levels are reached within 90 minutes.

Oxytocin is a neuropeptide which crosses the blood-brain barrier reliably after intranasal administration [21]. The spray was administered five times, 45 minutes before onset of tasks, with a delay of 45s between administrations. Each administration consisted of one insufflation of the spray into each nostril. Each inhalation contained approximately 4 international units (IU) (total: 40 IU) (procedure confer [22]). In between the three task blocks, participants received 2 ‘booster’ doses of oxytocin (each 8 IU) in order to keep steady state levels of oxytocin during testing. Oxytocin plasma levels peak after 15 minutes and decrease after 30–60 minutes (24–26 IU) (e.g. [21]). Behavioural testing is commonly conducted 45 minutes after administration [22]–[25].

### Procedures

Participants were requested to abstain from any drug use 1 week before the medical examination until the last test day. They were asked not to use any caffeinated or alcoholic beverages 24 h before testing and to get a normal night's sleep. Prior to experimental sessions at 9 AM they were screened for drugs of abuse consumption in urine (THC/opiates/cocaine/amphetamines/methamphetamines), and had to pass a breathalyser ethanol test. Women were given a pregnancy test. When tests were negative, participants had breakfast and filled out two questionnaires (to assess sleep complaints and their mood state). See Table 2 for a full description of a test day.

Test days were minimally separated by 7 days. Prior to test days, participants were familiarized with the tests on a training day. Participants provided written informed consent to participate in this study and were paid upon completion of the testing periods for their participation.

The study was performed in accordance with the Helsinki Declaration of 1975 (Latest revision, Seoul 2008) and was approved by the Medical Ethics Committee of the Academic Hospital of Maastricht and the University of Maastricht (Reference number: 11-3-001; NL34859.068.10).

### Empathy tests and questionnaire

#### Reading the Mind in the Eyes test (RMET)

The revised version of the RMET consists of 36 pictures of eye regions expressing a complex emotional state [26]. Pictures were shown subsequently on a computer screen, accompanied by 4 emotion words. Participants had to select the emotion word that matched the depicted emotion. The 36 pictures can be classified into 3 emotional valence categories: i.e. negative (N = 12), positive (N = 8), neutral (N = 16) [27]. Dependent variables are total number correct answers, the percentage of correct responses per valence category and corresponding response times. This task measures the ability to infer the mental state of others from social cues in the eye region or cognitive empathy and has been shown to be sensitive to the administration of oxytocin [25].

#### Multifaceted Empathy Test (MET)

The MET [28] consists of 40 pictures of people conveying a complex emotional state which was positive in 50% of the pictures and negative in the other half. To assess cognitive empathy (CE), participants had to select, out of 4 words, the emotion word that matched the emotion picture. To assess emotional empathy (EE), participants had to rate on a scale from 1–9 ‘how aroused this picture made them feel’ ( =  Implicit EE) and ‘how concerned they were for the person’ ( =  Explicit EE). Dependent variables are the number of correct classified pictures and corresponding reaction times and the IEE and EEE ratings per valence.

#### Interpersonal Reactivity Index (IRI)

The IRI is a 28-item questionnaire consisting of 4 discrete seven-item scales i.e., ‘Fantasy’, F (tendency to imaginatively transpose oneself into fictional situations), ‘Perspective-Taking’, PT (tendency to spontaneously adopt the psychological viewpoint of others), ‘Empathic Concern’, EC (taps the respondents' feelings of warmth, compassion and concern for others), and ‘Personal Distress’, PD (assesses self-oriented feelings of anxiety and discomfort resulting from tense interpersonal settings). The first two scales are a measure of Cognitive Empathy; the two latter a measure of Emotional Empathy [29]. The IRI, originally designed as a trait measure, was used in the present study as a state measure. The questions refer to feelings and thoughts experienced in different situations. To be able to measure the effect of state on these questions/scales, the original instructions were adjusted i.e. participants were asked to answer these questions keeping in mind the state they were in.

### Social interaction tests and questionnaire

#### Trust game

The Trust Game aims to assess the ability to infer the mental state of another and to cooperate in order to make beneficial choices [30]. This measure has been shown to be sensitive to the effects of oxytocin i.e. it causes a substantial increase in trust in healthy males [31].

In this computerized version of the trust game, participants were presented with one-off fixed two-choice money trials in which there were two players; i.e. the participant and another player. Both could either be in the role of player 1 ( =  the ‘trustor’) or player 2 ( =  the ‘trustee’). Participants were randomly assigned the role of ‘trustor’ and ‘trustee’ over subsequent trials. Participants were instructed that they were not playing directly with other players but that they played with the implementation of answers of players which were gathered in a previous experiment. They were explained that their decisions would have consequences for those other players and that payment of all players would take place after completion of the experiment (for similar methods see: [30], [32]).

Player 1 always had two options, i.e. to either trust player 2 or defect. In case he defected, the game ended and player 2 had to settle with the reward that he was given. When player 1 chose to give trust, player 2 had the possibility to either reciprocate that trust, in case both got an equal monetary reward; in case s/he exploited the trust, the decision was only be beneficial for player 2.

Two factors, Risk (high/low) for the ‘trustor’ and Benefit (high/low) for the ‘trustee’ were manipulated separately and both could affect trust and reciprocity decisions. The risk manipulation determined the risk for player 1. In the high-risk condition, player 1 could lose a large amount of money by trusting player 2, in case the latter chose to defect. In the low-risk condition, player 1 could lose only a small amount of money by trusting player 2. The benefit manipulation determined the benefit for player 2 when being trusted. In the low-benefit condition, the difference between money gained by player 2 when being trusted relative to not being trusted was small. In contrast, in the high-benefit condition, the increase of money for player 2 by being trusted was large. In total, the task consisted of 90 trials: i.e. 20 high-risk, high-benefit, 20 high-risk, low-benefit, 20 low-risk, high-benefit, 20 low-risk, low benefit and 10 no-risk, no-benefit trials. These additional manipulations enabled us to obtain a behavioral measure of perspective taking within the task (e.g. [33], [34]).

Dependent variables are percentage of Trust ( =  number of times trust is given/ (number times trust+ number times no trust)) and percentage of Reciprocity ( =  number of times trust is reciprocated/ (number times reciprocated+ number times not reciprocated)). Participants knew that they would get an actual reward (between €3–5) at the end of each game, based on the reactions they have given in the game.

#### Social Ball Tossing Game

The adapted version of the CyberBall task designed by Andari and collaegues (2010) [35] was used, i.e. the Social Ball Tossing Game. This is a social interaction task measuring the participants' choice of cooperative (good, bad, neutral) opponent player. This relatively new task has been used in oxytocin research with high functioning autism spectrum disorders showing improved social behavior in these participants [35]. In the Social Ball Tossing Game, participants played a tossing game with 3 other virtual (fictitious) opponents. Whenever they received the ball they had to throw it back to one of the three opponents. The profile of these players gradually change to that of a good (including), a bad (excluding) and a neutral player. The good player threw the ball back at the participant, whenever s/he threw the ball to the good player; the bad player excluded the participant from the game by not returning the ball. The neutral player threw to the other players equally. The participants were told they were playing this game online with 3 real humans. Dependent variables are the percentage of tosses to the good, bad and neutral player.

#### Social Ball Tossing Game Questionnaire (SBTGQ)

At the end of the Social Ball Tossing Game participants were asked to estimate, using a subjective seven-point rating scale, their sentiments of ‘trust’ and ‘preference’ with respect to the fictitious players [35].

### Control measures

#### Word Learning Task (WLT)

The WLT was added as an active control task, to prove the successfulness of the MDMA manipulation, as it has repeatedly demonstrated selective MDMA effects (e.g. [20], [36]). It consists of thirty Dutch mono-syllabic meaningful nouns (N = 18) and adjectives (N = 12) which were consecutively presented on a computer screen [20], [37]. The words are either neutral (N = 6) or have a valence (i.e. positive (N = 12) or negative (N = 12)). The words in the lists of the four parallel versions had been matched for abstraction. Participants had to recall verbally as many words as possible (immediate recall). This procedure was repeated 3 times; immediate scores were summed to comprise the Total Immediate Recall score. After a 30-minute delay participants were asked to recall as many of the previously learnt words as possible ( =  delayed recall). Hereafter, participants were given a delayed recognition task containing 15 new words and 15 words of the previously shown list. Participants' task was to indicate whether the presented word was a new one or one from the original list. Dependent variables are the number of correct recalled words per trial, Total Immediate Recall score, the Delayed Recall score, the Delayed Recognition score (total number correct items of the original list; max score = 15) and corresponding RTs.

#### Groninger Sleep Scale (GSS)

The GSS [38] assesses sleep quality and quantity (hours of sleep). It consists of fifteen dichotomous questions about sleep complaints and an open question concerning the duration of sleep. The number of hours sleep and the total score on this questionnaire were compared over the four test days to ascertain that participants had an equal amount of sleep quantity and quality before each test day.

#### Profile of Mood States (POMS)

The POMS [39] is a self-assessment mood questionnaire with 72 five point-Likert scale items, representing eight mood states; i.e. Anxiety, Depression, Anger, Vigor, Fatigue, Confusion, Friendliness and Elation. Two extra scales are derived, i.e. Arousal ((Anxiety + Vigor) – (Fatigue + Confusion)) and Positive mood (Elation - Depression). The participant had to indicate to which extent items were representing his/her mood.

#### National Adult Reading Test (NART)

The Dutch version of the NART was used to estimate the verbal intelligence of participants [40]–[42].

#### Physiological assessments

Heart rate and (dia/systolic) blood pressure were assessed three times on each test day and served as control measures to assure the participants were in good medical condition throughout the test day (See Table 2).

### Endocrine measures and Pharmacokinetics

Blood samples were collected three times on each test day in order to determine endocrine concentrations (oxytocin, cortisol) and pharmacokinetics of MDMA and MDA, or pindolol. See Table 2 for detailed sampling schedule.

#### Pharmacokinetics

Blood plasma samples were frozen at −20°C until analysis for drug concentrations. MDMA, MDA, HMMA and HMA were determined using a method previously described by Pizarro et al. (2002) [43]. Pindolol plasma concentrations were determined following a modified method of Sprake and Gibb (2007, Waters Corporation) using a HPLC-MS method (Bruker esquire 3000plus, Bruker Corporation, Billerica, MA). Briefly, daily standard curves were fortified with pindolol 0, 10, 25, 50, 75 and 100 ng·mL-1 using 200 µL of blank plasma. After liquid extraction and evaporation step, each sample was reconstituted with 100 mL of the mobile phase, 2 mM amonium acetate+0.1% formic acid in water-0.1% formic acid in acetonitrile (85∶15, v/v). Separation of pindolol and its internal standard (nadolol) was carried out using a 2.1×100 mm Waters Atlantis (Waters Corporation, Milford,MA) dC18 3 um column. The linear gradient elution system was as follows: 85% A for the initial time, rising to 5% A at the first 1.6 min, and then reached to 85% A at 2 min. The total running time was 3.2 min.

The flow rate was 0.3 mL·min -1. Identification of compounds was carried out by comparing retention times and UV spectra of the unknown peaks with those of the standards at 254 nm. LOD and LOQ were 0.5 and 1.5 ng·mL -1, respectively. Intra-and inter-assay variability was below 15%.

#### Endocrine measures

A 2-mL sample for hormone analysis (cortisol and oxytocin) was drawn and collected in nonheparinized tubes at 0 and at 90, 120, 150 and 165 min after drug administration. Samples were centrifuged at 3500 rpm for 10 min and at 4°C. Serum was removed and frozen at −80°C until analysis.

Cortisol samples were analysed with the AxSYM Cortisol Assay (Abbott Diagnostics, Abbott Park, IL) that utilizes fluorescence polarization immunoassay (FPIA) [44] according to the manufacturers' instructions. Serum oxytocin concentrations were determined by a fluorescent immunoassay kit (Phoenix Pharm. Inc, Burlingame, CA) following the manufacturers' instructions.

### Statistical analyses

#### GLM

Data entered a general linear model, repeated measures procedure (SPSS, version 18.0) with Treatment Condition (4 levels) as main within subject factor. Extra within-subject factors were included for the following assessments: WLT (Trial: 3 levels; Valence: 3 levels); RMET (Valence: 3 levels); MET (Valence: 2 levels); Trust Game (Risk: 2 levels; Benefit: 2 levels); Social Ball Tossing Game (Player Type: 3 levels). In case of main effects, contrast-analyses were conducted (Treatment-Placebo and MDMA vs pindolol + MDMA).

For the POMS and physiological measurements an initial GLM was conducted, including only baseline, to test for baseline differences. In case there were no differences, a second GLM was conducted including the second (POMS) or the second and third (physiological) measures.

Friedman's two-way analysis of variance by ranks was run on the endocrine levels as they were not normally distributed. The Wilcoxon matched-pair signed-rank test was conducted in order to find out which treatment was the source of the significance (Treatment-Placebo and MDMA- pindolol+MDMA differences).

#### Correlation analyses

In order to test the association between cortisol and oxytocin concentrations, Spearman's rho was calculated at three time points (baseline, before, and after test battery). To test whether behavioural responses (MET) corresponded with subjective perceptions (IRI) of empathy, both measured at peak drug concentrations, Pearson's r was calculated. To test the association between oxytocin concentrations and empathy responses (MET) Spearman's rho was calculated.

The alpha criterion level of significance for all analyses was set at p = 0.05; Holms sequential Bonferroni correction was applied for contrast analyses [45].

## Results

### Empathy tests and questionnaires

#### RMET

Analyses revealed a main Valence effect on percentage correct (p<.001) and corresponding response times (<.001). Further analyses revealed a significant difference between correct identification and response times of positive valence items compared with neutral items, i.e. participants were better and faster in identifying positive emotions compared with neutral expressions. There were no Treatment Condition effects on total number correct, percentage correct or corresponding response times (see Table 3).

**Table 3:** Mean (SE) of dependent variables (DV) of the empathy & social interaction measures and main effects of Treatment Condition and Task Variable* (Valence: MET; Player Type: Social Ball Tossing Game; Risk-Benefit: Trust Game); statistically significant treatment-placebo contrasts are flagged with an ‘a’; ns =  not significant, na =  not applicable.

	Treatment Condition (Mean ± SE)				GLM			
					Treatment Condition		Task variable*	
Tests/DV	MDMA	Pindolol + MDMA	Oxytocin	Placebo	F	p	F	p
**RMET**							Valence	
Total correct (#)	26.85 (.95)	26.60 (.87)	27.85 (.64)	27.55 (.65)	1.47	ns	na	na
Correct - (%)	66.67 (3.47)	66.67 (3.42)	72.92 (4.35)	69.17 (3.20)	1.65	ns	23.39	<.001
Correct + (%)	85.62 (3.18)	84.37 (3.38)	87.50 (2.56)	89.37 (2.27)				
Correct 0 (%)	75.00 (3.11)	74.06 (2.91)	75.62 (2.93)	75.62 (2.31)				
RT _correct_ – (msec)	5587.30 (514.143)	5212.28 (354.36)	6371.73 (459.73)	6010.49 (385.29)	1.04	ns	10.54	<.001
RT _correct_ + (msec)	4946.27 (465.87)	5051.72 (526.56)	4981.94 (324.10)	5367.53 (440.58)				
RT _correct_ 0 (msec)	5999.79 (444.51)	5419.69 (406.08)	6200.52 (433.41)	6270.49 (460.19)				
**MET**								
Cognitive Empathy							Valence	
Total correct (#)	23.90 (.80)	23.15 (.76)	23.80 (.91)	23.90 (.68)	.41	ns	na	na
Correct – (%)	55.75 (3.19)	55.00 (2.49)	57.25 (3.25)	54.25 (3.00)	.41	ns	6.53	.019
Correct + (%)	63.75 (2.40)	60.75 (2.21)	61.75 (2.57)	65.25 (1.93)				
RT _correct_ -	4329.30 (288.59)	4075.77 (252.52)	4768.94 (379.26)	4521.86 (296.12)	1.37	ns	39.24	<.001
RT _correct_ +	3834.17 (344.62)	3428.98 (241.42)	4091.71 (351.53)	3983.39 (238.48)				
Emotional Empathy (Explicit; ‘Concerned’)							Valence	
Rating all items	4.26 (.27)^a^	4.35 (.27)^a^	4.18 (.30)	3.94 (.27)	2.70	.054	na	na
Rating -	4.28 (.41)	4.29 (.38)	4.41 (.39)	4.23 (.37)	2.70	.054	.33	ns
Rating +	4.23 (.36)	4.40 (.39)	3.94 (.32)	3.66 (.30)				
Emotional Empathy (Implicit; ‘Aroused’)							Valence	
Rating all items	4.39 (.27)^a^	4.44 (.26)^a^	4.18 (.30)	4.02 (.27)	3.33	.026	na	na
Rating -	4.51 (.34)	4.58 (.34)	4.42 (.34)	4.28 (.33)	3.33	.026	2.01	ns
Rating +	4.26 (.31)	4.30 (.31)	3.94 (.31)	3.75 (.29)				
**IRI**								
PT	24.10 (.80)	24.91 (.74)	25.53 (1.10)	24.53 (.83)	1.26	ns	na	na
FS	22.11 (1.74)	23.42 (1.56)	22.37 (1.78)	22.16 (1.49)	.98	ns		
EC	23.41 (1.23)	26.61 (.69)^a^	25.42 (.87)^a^	24.26 (.71)	4.16	.010		
PD	15.63 (.89)	16.42 (.81)^a^	17.37 (.96)	14.26 (.91)	4.72	.005		
**Trust Game**							Benefit/Risk	
Trust (%)	56.30 (.06)	50.80 (.06)	56.00 (.06)	52.20 (.06)	1.65	ns	B: 7.58	.013
							R: 62.37	<.001
Reciprocity (%)	40.50 (.09)	38.90 (.09)	34.70 (.09)	34.50 (.09)	.47	ns	B: 2.86	ns
							R: 5.16	.035
**SBTG**							Player Type	
Tosses-Good (%)	49.79 (5.21)	47.77 (4.26)	48.34 (5.04)	45.30 (3.73)	1.00	ns	13.24	<.001
Tosses- Bad (%)	25.01 (3.41)	29.15 (3.63)	30.44 (3.80)	27.36 (2.93)				
Tosses-Neutral (%)	25.00 (3.31)	23.08 (2.17)	21.22 (2.28)	27.30 (2.26)				
**SBTG-questionnaire**								
Trust- Good	4.15 (.47)	5.12 (.37)	4.05 (.43)	4.70 (.44)	1.04	ns	na	na
Trust-Bad	3.95 (.39)	4.05 (.33)	4.57 (.42)	4.30 (.40)				
Trust-Neutral	4.20 (.47)	3.15 (.42)	4.07 (.29)	3.97 (.40)				
Preference-Good	4.15 (.53)	5.22 (.39)	4.02 (.51)	5.00 (.47)	1.07	ns	na	na
Preference-Bad	3.95 (.48)	3.97 (.33)	4.67 (.47)	4.10 (.44)				
Preference-Neutral	4.05 (.49)	3.20 (.48)	4.02 (.33)	3.97 (.42)				

#### MET

Analyses revealed an almost significant Treatment Condition effect on Explicit (p = .054) and a significant effect on Implicit (p = .026) Emotional Empathy. Further analyses showed that under the influence of pindolol+MDMA (p_explicit_ = .014; p_implicit_ = .007) or MDMA (p_explicit_ = .018; p_implicit_ = .007), participants felt more concerned and more aroused by the emotional content of the pictures compared with placebo. Both MDMA conditions did not differ significantly from each other, i.e. pindolol did not alter the MDMA effect. There was no effect of oxytocin on measures of emotional empathy. There was an effect of Valence on Cognitive Empathy (p = .019); participants identified more positive emotions correctly compared to negative ones. There was no effect of Valence on measures of emotional empathy and no effect of Treatment Condition on Cognitive Empathy, nor a Treatment Condition by Valence interaction (see Table 3).

#### IRI

Analysis revealed a main effect of Treatment Condition on scales assessing emotional empathy; i.e., Emotional Concern (p = .010) and Personal Distress (p = .005). Further analyses revealed that these effects were due to pindolol+ MDMA (p_EC_ = .006; p_PD_ = .012) and oxytocin (p_EC_ = .048; p_PD_<.001), causing higher levels of emotional concern and personal distress. The effect of oxytocin on Emotional Concern was not significant after Helm's Bonferroni correction. There were no Treatment Condition effects on scales assessing cognitive empathy, i.e. Perspective-Taking and Fantasy (see Table 3).

MET-IRI correlation analysis revealed associations between all the IRI scales (PT, F, EC, PD) and both types of emotional empathy (implicit, explicit) (r-range:.31–.42; p<.001–.005). There was no association between IRI scales and cognitive empathy as measured with the MET.

### Social interaction tests and questionnaire

#### Trust Game

Analysis revealed no main effects of Treatment Condition on Reciprocity or Trust. There was a main effect of Risk (p = .035) on Reciprocity, i.e. when the level of risk was high, i.e., when player 1 could lose a large amount of money by trusting player 2 ( =  the participant), there was more reciprocity by the participant. This means that participants did not violate the trust of the other player when a great loss for that player was at stake; this effect was independent of Treatment Condition.

There was a main effect of Risk (<.001) and Benefit (p = .013), and their interaction (p = .028), on Trust. The main effects showed respectively that when Risk was high, Trust was low, and when Benefit was low, Trust was low. However, it was also shown that effects of Risk and Benefit on Trust were interdependent, i.e. Trust was the lowest when Risk was high and Benefit low; when Benefit increases, Trust increases but it was still lower than Trust at low Risk levels. There was no interaction effect of Treatment Condition by Risk or Benefit on Trust (see Table 3).

#### Social Ball Tossing Game

Analyses revealed a main effect of Player (p<.001) on number of tosses. Participants tossed more balls to the good player compared with the neutral player. There was no main effect of Treatment Condition, or a Treatment Condition by Player interaction (see Table 3).

#### Social Ball Tossing Game Questionnaire

Analysis revealed a significant main effect of Player Type on both items of the questionnaire i.e. trust in other player (p = .022) and preference for player (p = .019). Further analyses revealed that, irrespective of Treatment Condition, there was more trust in, and preference for the ‘good’ player compared with the ‘neutral’ player. There was no main effect of Treatment Condition on items of the questionnaire (see Table 3).

### Endocrine measures and pharmacokinetics

Mean (SD) oxytocin and cortisol concentrations are listed in Table 4. Friedman's 2-way ANOVA revealed no significant differences between baseline oxytocin and cortisol levels on the 4 test days. Blood drawing was not always successful; the number samples included in the analysis is mentioned in Table 4.

**Table 4:** Oxytocin (pg/mL) and cortisol (nM or nmol/L) serum concentrations * B =  Baseline; Start of test battery = 45′post-oxytocin administration, 90′post-MDMA administration, 150′post-pindolol administration; End of test battery = 120′post-oxytocin administration (first dose), 165′post-MDMA administration, 225′ post-pindolol administration; Statistically significant treatment-placebo contrasts are flagged with an ‘a’, MDMA vs pindolol + MDMA contrasts with a ‘b’.

	Measurement*					
**Oxytocin**	B		Start of test battery		End of test battery	
Conditions	M(SD)	N	M(SD)	N	M(SD)	N
MDMA	6.47 (6.05)	17	12.07 (15.85)	18	7.47 (4.79)	16
Pindolol + MDMA	5.67 (3.88)	17	10.71 (11.39)	18	9.96 (6.22)	11
Oxytocin	5.42 (4.63)	14	12.85 (11.49)^a^	16	16.07 (12.62)^a^	12
Placebo	6.24 (5.64)	16	5.24 (6.25)	15	5.05 (4.58)	14
**Cortisol**	B		Start of test battery		End of test battery	
MDMA	704.90 (256.66)	18	735.10 (208.66)^a^	18	780.13 (361.10)^a,b^	17
Pindolol+MDMA	746.29 (311.36)	18	739.39 (246.35)^a^	18	799.05 (317.83)^a,b^	16
Oxytocin	653.20 (210.71)	17	351.92 (160.62)	18	323.04 (116.76)	11
Placebo	633.42 (178.76)	17	410.53 (154.66)	15	293.43 (129.77)	14

#### Oxytocin

Friedman's 2-way ANOVA of oxytocin levels revealed a Treatment Condition effect 90′post-MDMA/45′post-oxytocin (p = .034) but not 165′post-MDMA/120′post-oxytocin. Separate treatment-placebo contrasts revealed only a significant effect of oxytocin treatment (p = .033) on oxytocin levels; the other contrasts approximated significance: i.e. MDMA vs placebo (p = .071) and pindolol + MDMA vs placebo (p = .071). Oxytocin administration caused an increase of almost 2.5 times the oxytocin concentrations in the placebo condition. MDMA and pindolol + MDMA caused an increase of respectively 2.3 and 2 times placebo levels. The MDMA-induced cortisol increment in the pindolol-MDMA condition was unaffected by pindolol as shown by an absence of significant differences between both MDMA conditions. For explorative reasons, treatment-placebo contrasts were also conducted for the third sampling moment, i.e. 165′post-MDMA/120′post-oxytocin. This analysis revealed a significant effect of oxytocin on oxytocin levels (p = .021); the MDMA-placebo contrast approximated significance (p = .052).

#### Cortisol

Analysis revealed a significant Treatment Condition effect on cortisol levels 90′ (p<.001) and 165′post-MDMA/120′post-oxytocin (p<.001). Further analysis revealed that both effects were caused by the MDMA (p = .001; p<.001) and the pindolol + MDMA (p<.001; p = .001). During both Treatment Conditions, cortisol levels increased 1.8 and 2.7 times placebo-cortisol levels, respectively 90 and 165 minutes post-MDMA. MDMA-induced cortisol increments did not differ between the MDMA-only and pindolol+ MDMA condition at 90′ post-MDMA; they did differ at 165′ post-MDMA administration, i.e. cortisol concentrations were slightly higher in the ‘combined’ condition compared with the MDMA-only condition (p = .008). Oxytocin administration did not affect cortisol levels.

#### Cortisol-oxytocin concentration correlations

Spearman's rho revealed significant positive correlations between cortisol and oxytocin levels at baseline (r_s_ = .45, p<.001; N = 64) and 90′post MDMA (r_s_ = .28, p = .022; N = 67). There was no significant correlation between hormone levels at 165′post-MDMA/120′post-oxytocin (N = 50).

#### Oxytocin-Emotional Empathy (MET) correlations

Spearman's rho did not reveal significant correlations between oxytocin concentrations and emotional empathy (i.e. EEE-negative (r_s_ = −.037), EEE-positive (r_s_ = .133), EEI-negative (r_s_ = .015), EEI-positive (r_s_ = .068)).

#### Pharmacokinetics

Mean (SD) MDMA and MDA plasma concentrations (µg/L) were 127.65 (45.66) and 4.23 (2.33), 90 minutes post-MDMA administration and 141.68 (65.03) and 6.58 (2.34) 165 minutes post-MDMA administration. When combined with pindolol, mean (SD) MDMA and MDA plasma concentration were 105.67 (40.50) and 3.57 (2.46), 90′ post-MDMA administration and 131.57 (42.23) and 6.61 (2.31) 165′ post-MDMA administration (Table 5). Paired t-tests revealed a significant difference in MDMA concentrations between the MDMA alone and pindolol+MDMA condition (t(15) = 2.3; p = .036). Pindolol pretreatment resulted in lower MDMA concentrations, only 90′post-MDMA; this difference was not present at 165′ post-MDMA.

**Table 5:** Mean (SD) MDMA/MDA concentrations (µg/L) in MDMA and the combined pindolol+MDMA condition.

	MDMA Condition				Pindolol + MDMA Condition			
	90′ post-MDMA		165′ post-MDMA		90′ post-MDMA		165′ post-MDMA	
	MDMA	MDA	MDMA	MDA	MDMA	MDA	MDMA	MDA
Mean	127.65	4.23	141.68	6.48	105.67	3.57	131.57	6.61
SD	45.66	2.33	65.03	2.34	40.50	2.46	42.23	2.31
N	17		17		18		16	

Mean (SD) pindolol plasma concentrations were 105.91. (23.67) and 116.69 (26.87) respectively 150′ and 225′ post-pindolol administration (Table 5).

### Control measures

#### WLT

Due to technical failure, responses of 1 participant during the recognition task are missing; therefore 19 participants are included in that analysis.

Analyses revealed significant main effects of Treatment Condition (p = .008) and Trial (p<.001) and an interaction effect of Treatment Condition by Trial (p = .040) on immediate recall scores. The Trial effect reflects the increase in number of correct recalled words over the three subsequent trials. Further analysis of the Treatment Condition effect reflected a significant difference between pindolol+ MDMA and placebo (p = .020). While under the influence of the combined treatment, participants recalled 5.80 words less in total compared with placebo. Additional analysis of the Treatment Condition by Trial interaction indicated that while performance under influence of oxytocin and placebo followed a linear course, increasing gradually over the three trials, performance under influence of pindolol+MDMA or MDMA, followed a more curved line. The latter meaning that performance increased linear from trial 1 to 2, and in line with placebo (though lower), while recall stagnated from trial 2 to 3; Participants under influence of MDMA (i.e. with or without pindolol) recalled 3.05 words less compared with placebo on trial 3.

Delayed recall scores revealed a significant Treatment Condition effect (p = .001). Further analysis showed that participants recalled on average 4.15 and 3.15 words less under influence of pindolol+ MDMA with (p = .001) or MDMA (p = .007), relative to placebo.

There were no effects of Treatment Condition on total correct recognized items or on corresponding reaction times (Table 6).

**Table 6:** Mean (SE) of dependent variables of the WLT and main effects of Treatment Condition and Task Variable* (Trial); ns =  not significant, na =  not applicable.

	Treatment Condition (Mean ± SE)				GLM			
					Treatment Condition		Task variable*	
Tests/DV	MDMA	Pindolol + MDMA	Oxytocin	Placebo	F	p	F	p
**WLT-30 IR/DR**							Trial	
IR_T1_	8.45 (.74)	8.20 (.53)	10.20 (.55)	9.70 (.58)	4.35	.008	77.25	<.001
IR_T2_	12.40 (.96)	11.60 (.67)	13.85 (.73)	12.80 (.78)				
IR_T3_	13.40 (1.17)^a^	13.30 (.96)	16.05 (1.05)	16.40 (.93)				
IR_TOT_	34.25 (2.71)	33.10 (2.00)	40.10 (2.05)	38.90 (2.04)	4.35	.008	na	na
DR	9.00 (1.05)^a^	8.00 (.85)^a^	11.15 (1.14)	12.15 (1.24)	6.45	.001	na	na
**WLT-30 Recognition**								
Total correct (#)	12.21 (.50)	12.28 (.42)	12.68 (.42)	12.32 (.59)	.34	ns	na	na
RT _correct_ (msec)	753.15 (37.36)	743.52 (31.04)	762.62 (26.63)	762.71 (27.67)	.16	ns	na	na

#### GSS

There was no difference in sleep quality and quantity over the four test sessions. Participants slept on average 7.14h (SD = .93) and had and average score of 2.19 (SD = 2.71) on the GSS.

#### POMS

There were no baseline differences in POMS scores over the four test sessions. Analyses at peak concentrations of Treatment revealed main effects of Treatment Condition on 5 out of 10 scales of the POMS i.e. Anxiety (p = .001), Vigor (p = .012), Confusion (p = .000), Elation (p = .020), and Arousal (p = .003). Participants were more anxious (.001), vigorous (.018), confused (.001), elated (.008), and aroused (.020) while under influence of MDMA, compared with placebo. When MDMA was combined with pindolol, participants were also more anxious (.021), vigorous (.031) and aroused (.005) compared with placebo cf. the MDMA alone condition, but there was no effect of MDMA on Confusion and Elation when combined with pindolol, compared with placebo (see Table 7). Additional contrasts (MDMA- pindolol+MDMA) for Confusion and Elation revealed that pindolol did not alter the effects of MDMA on Elation but it did alter the effects on Confusion (p = .002), i.e. by lowering the incremental influence of MDMA on Confusion.

**Table 7:** Mean (SE) of subscales of the POMS and main effect (GLM) of Treatment Condition; T =  Time of measurement (B =  baseline; Peak =  drug peak moment); Statistically significant treatment-placebo contrasts are flagged with an ‘a’, MDMA vs pindolol + MDMA contrasts with a ‘b’.

		Conditions (Mean ± SE)				GLM	
Mood Scales	T	MDMA	Pindolol +MDMA	Oxytocin	Placebo	F	p
Anxiety	B	2.95 (.37)	2.35 (.34)	2.70 (.43)	2.85 (.46)	.65	ns
	Peak	7.40 (1.39)^a^	5.65 (1.16)^a^	2.90 (.45)	2.65 (.39)	7.78	<.001
Depression	B	.50 (.31)	.80 (.39)	.95 (.42)	1.10 (3.8)	.71	ns
	Peak	1.40 (.92)	1.05 (.44)	.45 (.20)	1.40 (.59)	.90	ns
Anger	B1	1.80 (.52)	1.85 (.58)	1.60 (.42)	2.10 (.58)	.26	ns
	Peak	1.20 (.45)	1.40 (.39)	1.30 (.29)	1.75 (.53)	.40	ns
Vigor	B	13.80 (.98)	11.60 (1.13)	11.90 (1.03)	11.90 (.93)	1.91	ns
	Peak	13.90 (1.39)^a^	13.00 (1.18)^a^	11.40 (1.10)	9.80 (1.07)	4.02	.012
Fatigue	B	1.40 (.45)	1.80 (.42)	1.90 (.80)	1.95 (.55)	.38	ns
	Peak	.85 (.32)	1.05 (.60)	1.65 (.54)	2.10 (.82)	1.59	ns
Confusion	B	3.20 (.29)	3.50 (.33)	3.65 (.42)	3.60 (.42)	.73	ns
	Peak	8.10 (.95)^a, b^	5.60 (.70)^b^	4.80 (.47)	4.65 (.52)	8.45	<.001
Friendliness	B	18.90 (.85)	17.55 (.95)	17.55 (.98)	17.40 (1.04)	1.07	ns
	Peak	19.40 (1.27)	17.95 (1.22)	17.25 (1.31)	16.00 (1.20)	2.58	ns
Elation	B	10.55 (.55)	9.65 (.62)	9.65 (.75)	9.15 (.75)	1.52	ns
	Peak	12.15 (.87)^a^	10.85 (1.08)	9.90 (.85)	8.70 (.76)	3.53	.020
Arousal	B	12.15 (1.21)	8.65 (1.39)	9.05 (1.70)	9.20 (1.23)	2.35	ns
	Peak	12.35 (1.88)^a^	12.00 (1.81)^a^	7.85 (1.27)	5.70 (1.63)	5.23	.003
Positive Mood	B	10.05 (.57)	8.85 (.77)	8.70 (.94)	8.05 (.99)	2.13	ns
	Peak	10.75 (1.29)	9.80 (1.28)	9.45 (.94)	7.30 (1.12)	2.18	ns

#### Physiological assessments

Analyses over baseline physiological parameters did not reveal statistically significant differences. Mean (± SE) blood pressure (BP) and heart rate (HR) values were: 114.57 mmHg ±3.28 for systolic BP, 71.21 mmHg ±2.52 for diastolic BP, and 73 bpm±2.69 for HR.

The second ANOVA including Measurement 2 and 3, respectively pre-test at peak treatment concentrations and post-test, revealed a main effect of Treatment Condition on systolic (p<.001) and diastolic (p<.001) BP and HR (P<.001). There was also a significant main effect of Measurement (p = .004) on systolic BP. The latter reflected a decrease in systolic BP of 5.22 mmHg from pre-test to post-test measurement. The former reflected a significant increase of 9.22 mmHg in systolic BP and of 7.82 mmHg in diastolic BP after treatment with MDMA, with or without pindolol, and an increase of 17.13 bmp for HR only after treatment with MDMA compared with placebo. An additional contrast (MDMA- pindolol+MDMA) for systolic and diastolic BP showed that pindolol partially countered the incremental effects of MDMA on systolic BP (p = .001) but not on diastolic BP. The values were within normal ranges (i.e. BP 140/90 and HR: 60–100 bpm).

## Discussion

The aim of the study was to assess the effects of MDMA on different objective and subjective measures of empathy and social interaction, and to investigate the roles of oxytocin and the 5-HT_1A_ receptor in these effects. Results showed that MDMA selectively affected emotional empathy and left cognitive empathy, trust and reciprocity unaffected. When combined with pindolol, these effects remained unchanged. Oxytocin did not affect objective measures of empathy and social interaction; however, oxytocin increased participants' perception of emotional empathy as measured by the IRI questionnaire. MDMA's effects on control measures (WLT, POMS) were in line with previous findings from the same group (e.g. [19], [36]).

It was shown that participants under the influence of MDMA, irrespective whether it was combined with pindolol or not, were more concerned and aroused when confronted with pictures of people with a positive or negative emotional expression. Cognitive empathy, defined as inferring complex emotions either for eye-pairs or full body pictures, was not affected by any treatment. Together these results suggest a selective role for MDMA in emotional empathy which is in line with previous findings from Hysek and colleagues (2013) [9]. The absence of effects of MDMA on cognitive empathy (positive and negative emotions) as measured with the RMET and MET were also in line with previous studies [7], [8]. When taking Valence into account in the RMET, Hysek and colleagues (2012) revealed dissociative effects of MDMA on cognitive empathy, i.e., increased accuracy in recognition of positive emotions and decreased accuracy in recognition of negative emotions. This finding was not replicated by the present study. Participants in our study were faster and more accurate at identifying positive emotions, compared to negative and neutral emotions; this effect was not influenced by the treatment they received. The performance level in the RMET of participants in the present study was comparable to other studies using healthy controls of comparable age [25], [26], [46] and higher compared to Hysek et al.'s participants (2012) who only reached the same levels under influence of MDMA [8]. It can be suggested that there was no room for improvement in our participants.

These findings on objective measures in the present study were only partly supported by the subjective measurement of empathy (IRI). It revealed that participants under influence of oxytocin, and MDMA, when combined with pindolol, rated themselves higher on emotional concern and personal distress compared to placebo. However, as the IRI is actually a trait measure, it was expected that these effects were smaller.

There were no effects of MDMA with or without pindolol or oxytocin on social interaction measures (trust & reciprocity) approximately 2 hours after MDMA administration (1 h15 minutes after oxytocin administration) as measured by a trust game and a virtual social ball tossing game. The latter paradigm was also used by Frye et al (2014) however in another version [12]. Whereas they had two games of 3 to 4 minutes in which social acceptance and social rejection were simulated by means of manipulating the number of throws the participant received (acceptance: received more than at chance level; rejection: received less balls than expected at chance level). Their main dependent variables were self-rated mood, self-esteem and perceived number of received throws. They showed MDMA showed decreased the negative effects of social rejection on subjective ratings of mood and self-esteem and increased the perceived percentage of received throws. We used a version of the social ball tossing game, previously designed by Andari and colleagues [35]. The game started with 3 virtual characters whose reciprocating behavior towards the participant changed over the course of the game to become either including, excluding or neutral, i.e. throwing more, less or an equal amount to the participant compared to the other (fictitious) participants. Participants were told they were playing online against three other humans, not a computer. The game took approximately 20 minutes and the dependent variable was the number of throws to the three types of players. We showed that participants preferred interaction with the ‘good’/including player over the other, which is in line with a previous study using the same paradigm [35]. The pattern of interaction was not differently affected by treatment. Subjective ratings of trust in and preference for the good player mirrored the objective parameters, i.e. participants expressed more trust in and preference for the good player; their ratings were unaffected by treatment. In contrast to Frye et al (2014), our participants were not impaired at reading the intention of others (e.g. including or excluding them). This might be due to the fact that they received more contextual information which they could compare (i.e. 3 distinctive profiles) instead of only one type of player in one game (either including or excluding them). Our participants had the choice to interact with the player they preferred and our data suggest that MDMA (or oxytocin) does not influence their primary choice compared to placebo. This situation is contrary to the one in the paradigm of Frye et al. (2014) where participants had no influence over the situation but were subjected to one. In addition, Frye et al. (2014) used indirect measures that concerned the inner state of the participant (i.e. mood, self-esteem) and suggest an inward focus; the current study used an objective measure of interaction (ball tosses), complemented with measures about feelings towards the other (trust/preference) i. e. an outward focus.

The Trust Game was the second social interaction measure. It was shown that participants did not violate the trust of the other participants when a lot of money was at stake. This higher level of reciprocity in a high-risk context relative to a low-risk context is according to Bos et al. (2011) a reflection of the recognition of the positive intentions of the person giving trust, i.e. a measure of social perspective taking (also cognitive empathy) [33]. This measure was not affected by treatment (MDMA/oxytocin). Reciprocity of trust is important for social interaction and it depends on individual differences in social value orientation (SVO) [34]. SVO is defined as ‘behavior that maximizes the sum of resources of the self and others and minimizes the difference between the two’. Hysek and colleagues (2013) assessed SVO and showed that 4 hours after administration of MDMA, especially male participants chose the ‘altruistic’ option in which more resources were allocated to the other person, compared to placebo. Their behavior was comparable to female behavior in the placebo condition. Their results revealed a sex-specific effect of MDMA on SVO. We did not look into the effects of sex on trust or reciprocity as power did not allow us to conduct this kind of analysis. It can be speculated that any effect of MDMA on trust/reciprocity in the present study was masked by the mixed-sex sample. The results of Hysek and colleagues (2013) indicate that it is very important to address this in any future MDMA study.

The second part of the aim of the present study was to investigate the roles of oxytocin and the 5-HT_1A_ receptor in the MDMA-induced ‘prosocial’ effects. Contrary to our expectations, pindolol did not block MDMA effects, and oxytocin administration did not mimic MDMA effects. First, it cannot be concluded from the present study that the 5-HT_1A_ receptor does not play a role in the MDMA effects. While pindolol blocks approximately 40% of both auto- and post-synaptic receptors, 60% can still be stimulated by MDMA. Previously, studies have shown the ability of pindolol (20 mg) to affect subjective mood states [17], [19], although limited evidence for pindolol by MDMA interaction effects were demonstrated [17], [19], [36], [47]. The two studies that showed an interaction effect, demonstrated this on subjective and physiological measures, and it was speculated that these effects could perhaps be attributed to the beta-adrenergic effects of pindolol, rather than its effects on the 5-HT_1A_ receptor [17], [47]. They also ascribed this relative absence of interactions to the relatively high dose of MDMA which was possibly more potent than the dose of pindolol. In the present study, pindolol did seem to exert some counteracting effects on the subjective and physiological control measures. It prevented MDMA-induced confusion, and increase in heart rate. It could be suggested that the subjective and physiological effects are caused by the normalization in heart rate and thus perhaps due to beta-adrenergic activity; however, elevated ratings of anxiety, vigor and arousal were still present, i.e. not counteracted by pindolol.

With regard to oxytocin, it can be determined that our oxytocin concentrations 90′ after MDMA (75 mg) administration (Δ baseline = 5.6 pg/mL) were approximately 10 times lower than the concentrations Hysek et al (2013) report, 1 to 2 hours after MDMA (125 mg) administration, i.e. 60 pg/mL. They were 3 times lower than the concentrations Dumont and colleagues (2009) report (34.3 pmol/L), 110 minutes after MDMA (100 mg) administration. Despite the low concentrations of oxytocin compared to studies of Dumont and Hysek respectively, we did show an effect of MDMA on emotional empathy. It can be suggested that higher doses of MDMA lead to higher oxytocin concentrations but it is questioned whether these oxytocin concentrations are at the base of behavioral effects. The oxytocin concentrations 45′ after oxytocin administration in the present study (12.85 pg/mL) were comparable to those after MDMA administration (12.07 pg/mL); while oxytocin administration did not lead to behavioral changes, MDMA did. Correlational analyses were unable to detect a relationship between behavioral effects and oxytocin concentration. This is in line with Hysek and colleagues (2013) who showed that endocrine measures did not correlate with behavioral measures.

In the present study, the effects of intranasal oxytocin administration were assessed between 45′ and 120′ post-administration of the first dose (40 IU). It has been stated that consistent effects of oxytocin can only be expected reliably in a time window of less than 90′, and in order to capture the main behavioural effects, testing should start about 20 to 30 minutes after oxytocin administration [48]. However, in the present study, 2 booster doses of intranasal oxytocin were given to prevent the decline in levels. Apparently these booster doses seem to have helped as the oxytocin concentrations in blood were even higher at the end of the test battery compared to the beginning (16.07 vs 12.85 pg/mL), and higher than those of Gossen et al. [48] (C_max_ = 5.5 pg/mL 30′after 26 IU) who suggested this short test interval. Striepens et al. (2013) also showed that there is a lag between oxytocin levels in CSF (significant increases at 75′) and those in blood after intranasal application of oxytocin (C_max_ at 15′ after 24 IU). They suggest that there is therefore some support for considering delaying starting tasks following 24 IU a little longer than 45 minutes [21]. In the present study, tests were conducted between 45′–120′ post-oxytocin administration. While the presence of an oxytocin effect on the IRI questionnaire approximately 1 h after administration supports this suggestion, alternative explanations besides the interval between administration-tests could explain the lack of oxytocin effects in the present study.

While animal research previously has shown that both oxytocin administration as well as stimulation of the 5-HT_1A_ receptor can lead to enhanced social behavior, comparable to that produced by MDMA administration [16], [49] findings of the present study do not seem to be in line with those of animal studies. However, some remarks are needed. First, social behavior and cognition are more complex in humans compared to animals. Secondly, the paradigms used in animal research approximate more the natural setting and might be a better indication of ‘natural’ social behavior than measured with computer paradigms, in humans. Besides the use of the ‘right’ paradigm, setting is known to play an important role in the subjective experience of MDMA. Outside the lab, MDMA use is associated with a social context and social expectations (e.g. ‘being loved’) about the effects and they act on those expectations (interactions with others) [2], [50]. In oxytocin research it is also suggested that the effects of oxytocin administration are very context-dependent [51], [52]. To illustrate, apparently a salient social context (e.g. contact with the other participant) is essential to elicit behavioural effects of oxytocin in a trust game [53]. In addition, the effects of intranasal oxytocin administration might be relatively small during the experience of emotional stimuli due to internal oxytocin release in the placebo condition, caused by the stimuli [51]. Furthermore, ‘set’ can also be of influence, i.e. there is a possibility that oxytocin effects depend on the initial emotional state of the individual [52]. Bartels (2012) put forward that ‘we need to find clever ways to present social stimuli in truly social context if we want to understand the complex ways in which oxytocin shapes the human nature’ [51].

Potential underlying mechanisms that are worth looking into in humans are the 5-HT_2A_ receptor and the V1A receptor (V1_A_Rs). Both receptors have been shown to play a role in prosocial behaviour in animal research. When the V1_A_Rs were blocked, enhancement of social behavior by oxytocin, vasopressin, or MDMA, was prevented [49]. The 5-HT_2A_ receptor was shown to be reduced in MDMA-treated animals, which was accompanied by a decrease in social behaviour [54]. In humans, it has been shown that blocking of this receptor prevented MDMA-induced positive affect, suggesting a role for this receptor in prosocial behaviour [19]. The latter is currently being investigated by our group.

A potential limitation of the present study is that only single doses of the each drug were given and it is known that different doses can produce different subjective and behavioural effects (e.g. [4], [7]). However, this approach is not unusual (e.g. [9]) but the information provided is dose-restricted and not generalizable. The dose of MDMA used in the present study, i.e. 75 mg equals a 1.07 mg/kg dose, which lies in the range usually used in this type of research e.g.: 0.75–1.89 mg/kg [4], [7], [9], [6] but more to the lower end. It is possible that slightly higher doses result in more pronounced MDMA effects on measures of empathy and social interaction as shown by previous research (e.g. [4], [7], [9], [6]). A second potential limitation is the use of peripheral measures of oxytocin as it has been shown recently that there is a substantial lag of 60 minutes between the oxytocin peak at peripheral level and that at central level [21]. The test battery in the present study was conducted between 45′–120′ post-oxytocin administration and therefore it is reasonable to assume that the measurements took place at the time when oxytocin concentrations reached their peak at central level. However, although the time frame of behavioural measures was presumably adequate, it has been shown that plasma and CSF oxytocin concentrations do not correlate on coincident sampling moments and that CSF concentrations were higher than plasma concentrations [21]. Therefore, the plasma concentrations in the present study are potentially an underestimation of central levels.

In sum, it can be concluded that a single dose of 75 mg of MDMA selectively enhances emotional empathy in humans. While the underlying neurobiological mechanism is still unknown, it is suggested that peripheral oxytocin does not seem to be the main actor in this. Future research should take contextual factors into account while assessing MDMA-induced prosocial effects and look into the roles of the serotonin 2A receptor and the vasopressin 1A receptor.
